# A comprehensive review of long non-coding RNAs in the pathogenesis and development of non-alcoholic fatty liver disease

**DOI:** 10.1186/s12986-021-00552-5

**Published:** 2021-02-23

**Authors:** Arezoo Gowhari Shabgah, Fatemeh Norouzi, Mahdiyeh Hedayati-Moghadam, Davood Soleimani, Naseh Pahlavani, Jamshid Gholizadeh Navashenaq

**Affiliations:** 1 School of Medicine, Bam University of Medical Sciences, Bam, Iran; 2 Department of Food Hygiene, Faculty of Veterinary Medicine, Shahid Chamran University of Ahvaz, Ahvaz, Iran; 3 Department of Physiology, School of Medicine, Jiroft University of Medical Sciences, Jiroft, Iran; 4 Department of Nutritional Sciences, School of Nutrition Sciences and Food Technology, Kermanshah University of Medical Sciences, Kermanshah, Iran; 5 Social Development and Health Promotion Research Center, Gonabad University of Medical Sciences, Gonabad, Iran; 6 Noncommunicable Diseases Research Center, Bam University of Medical Sciences, Bam, Iran

**Keywords:** NAFLD, NASH, LncRNA, Fatty liver, Non-coding RNA

## Abstract

One of the most prevalent diseases worldwide without a fully-known mechanism is non-alcoholic fatty liver disease (NAFLD). Recently, long non-coding RNAs (lncRNAs) have emerged as significant regulatory molecules. These RNAs have been claimed by bioinformatic research that is involved in biologic processes, including cell cycle, transcription factor regulation, fatty acids metabolism, and-so-forth. There is a body of evidence that lncRNAs have a pivotal role in triglyceride, cholesterol, and lipoprotein metabolism. Moreover, lncRNAs by up- or down-regulation of the downstream molecules in fatty acid metabolism may determine the fatty acid deposition in the liver. Therefore, lncRNAs have attracted considerable interest in NAFLD pathology and research. In this review, we provide all of the lncRNAs and their possible mechanisms which have been introduced up to now. It is hoped that this study would provide deep insight into the role of lncRNAs in NAFLD to recognize the better molecular targets for therapy.

## Introduction

One of the most prevalent causes of chronic liver disease is non-alcoholic fatty liver disease (NAFLD), which covers a histological spectrum of liver diseases ranging from simple steatosis to non-alcoholic steatohepatitis (NASH), and subsequently can lead to fibrosis, cirrhosis, and liver cancer [1, 2]. NAFLD is considered a global health problem in which the prevalence of this disease has been reported in 25% of adults worldwide [3, 4]. NAFLD directly relates to metabolic syndromes, including obesity, insulin resistance, and dyslipidemia, which are also known as the hepatic indicator of metabolic syndrome [5]. Type 2 diabetes, alcohol, and obesity are the primary risks, while hepatitis C and some medications such as glucocorticoids are the other risk factor for NAFLD [6].

NAFLD is now considered an independent cardiovascular disease’s risk factor so that cardiovascular disease being the foremost cause of premature death in these patients [7]. Diagnostic procedures for NAFLD include liver biopsy, ultrasound, liver function tests, and liver elastography [8, 9]. Despite the high burden of NAFLD on the community, no effective treatment has been provided so far, and today, it has become a major public health challenge worldwide [10]. General NAFLD management is based on the treatment of liver-associated metabolic disorders such as obesity, dyslipidemia, insulin resistance, and lifestyle modifications, including diet and physical activity for slow weight loss. Primary drug therapy aimed to improve liver status is only recommended for patients with non-alcoholic steatosis or hepatic fibrosis [11, 12]. To date, the molecular and cellular mechanisms involved in steatosis have not been fully understood. Still, evidence suggests that various factors are involved in the development and progression of steatosis to steatohepatitis and liver fibrosis, including adipose tissue inflammation, hepatic lipogenesis, insulin resistance, lipotoxicity, oxidative stress, and hepatic mitochondrial dysfunction [13–18]. In the pathogenesis of NAFLD, endoplasmic reticulum stress, lipogenesis, and inflammation play pivotal roles in the disease development [19, 20]. NAFLD is a multifactorial disease whose genes play an essential role in the susceptibility of individuals, and according to Genome-wide association studies (GWAS), the role of several genes to increase the risk of NAFLD in certain populations have been shown, of which *PNPLA3* and *TM6SF2* gene polymorphisms have a strong association with NAFLD [21]. It has been discovered many genes and pathways in the pathogenesis of NAFLD, including binding immunoglobulin protein (*BIP*), inositol-requiring transmembrane kinase/endonuclease (*IRE*), C/EBP homologous protein (*CHOP*), X-binding protein (*XBP*), acetyl–coenzyme A carboxylase enzyme (*ACC*), sterol regulatory element binding proteins 1c (*SREBP-1c*), lipoprotein lipase (*LPL*), stearoyl–coenzyme A desaturase 1 (*SCD1*), fatty acid synthase (*FASN*), tumor necrosis factor-α (*TNFA*), and monocyte chemoactive protein 1 (*MCP1*) [19].

A group of RNA molecules with a length of more than 200 nucleotides entitled long non-coding RNAs (lncRNAs) without any capacity to be translated into proteins have attracted countless attention in recent researches, especially for their roles in liver diseases [22, 23]. Primary researches into lncRNA have noted that they are conserved in sequence. However, there have been several challenges to define the different classes of lncRNAs, including lncRNAs with highly sequence conserved, lncRNAs with a portion of the transcript is conserved (e.g., splice sites and 5′ end), and lncRNAs transcribed from conserved synteny region of the genome but have no identical sequence [24]. Like mRNAs and microRNAs, RNA polymerase II transcribes lncRNAs. Most lncRNAs, after transcription by RNA polymerase II, are processed like mRNAs, including 3′-end polyadenylation, 5′-end-capping, introns splicing, and intracellular transport. Although several lncRNAs have small open-reading frames (ORFs), these ORFs may not encode any proteins [25].

LncRNAs are best acknowledged for their transcriptional regulatory functions, and their role as regulators of gene transcription is well recognized. Many experiments have demonstrated that some lncRNAs mediate gene activation or silencing and are associated with chromatin modification enzymes. To take a concrete example, the lncRNA XIST (X-inactive specific transcript) is transcribed from one chromosome-X in female cells and deactivates the other chromosome-X by employing polycomb repressive complex 2 (PRC2) during X-liked dosage compensation [26].

In contrast, small groups of lncRNAs have recently been described as post-transcriptional regulators of the gene. In this regard, lncRNAs have the potential to inhibit and promote the post-transcriptional processes of mRNA, including degradation, splicing, and translation. To mention or cite one example, lincRNA-p21 by recruitment of Rck (a translation repressor protein) and partial base-pairing has been shown to suppress the translation of JunB and β-catenin encoding mRNAs [27].

**Fig. 1:**
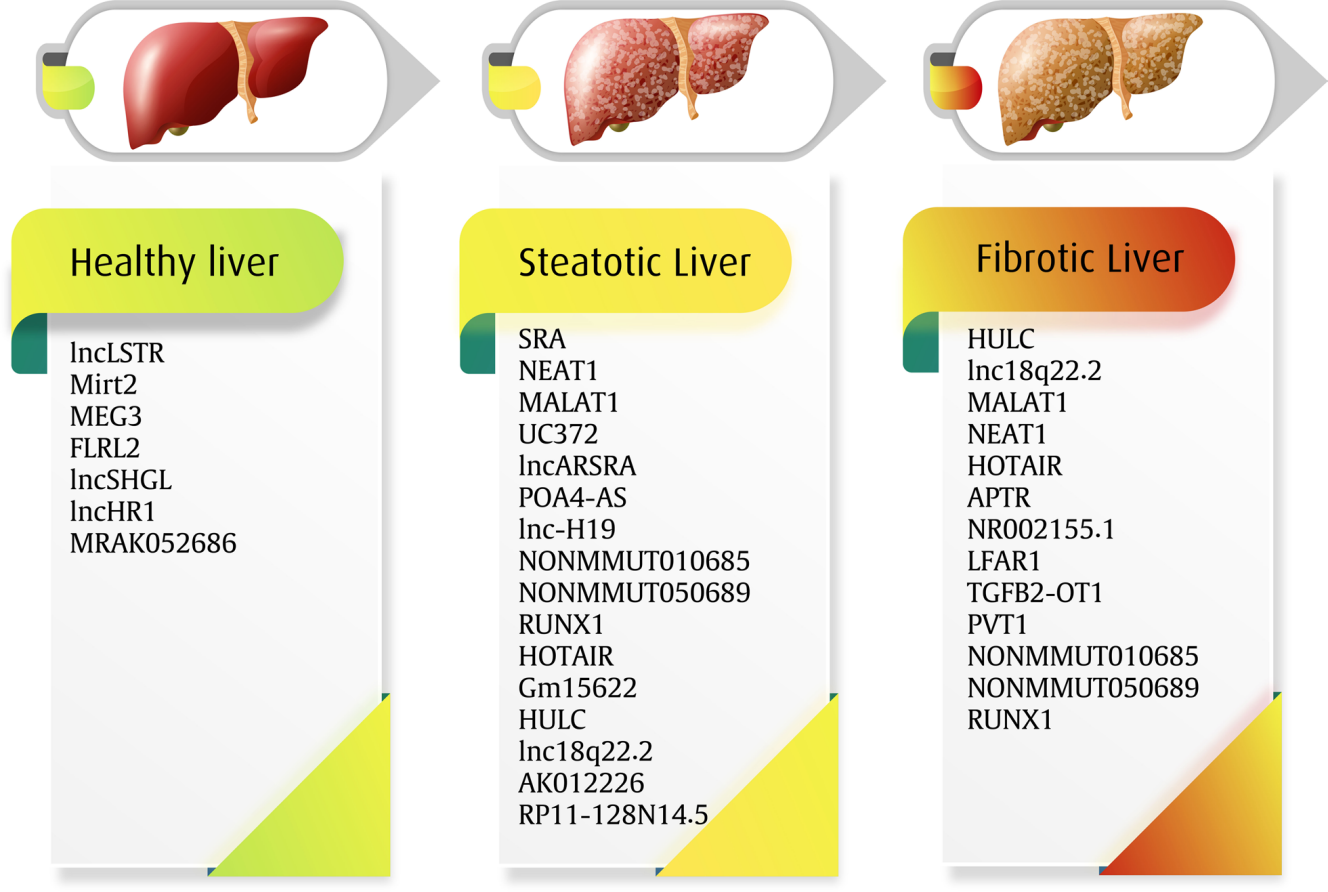
Schematic overview of the role of lncRNAs in fatty acid-related liver disease. This illustration depicted three groups of lncRNAs that progress the development of NAFLD, NASH, and hepatic fibrosis. All of the mentioned lncRNAs in three groups have been upregulated in the mentioned conditions. Therefore, it could be proposed the therapeutic panel to target elevated lncRNAs in the disease stages. Besides, this panel might be considered as the diagnostic panel in future studies. However, it should be mentioned that more studies are needed to determine their role in the disease

Previous studies have shown that lncRNAs have a crucial role in transcriptional and epigenetic regulation. Since lncRNAs likely influence the susceptibility to NAFLD [28], the purpose of the present review study is examining the role of lncRNAs in the pathogenesis and development of NAFLD (Table 1; Fig. 1).

**Table 1:** The summery of potentiating and preventing role of lncRNAs in fatty liver disease

lncRNA	Target	Role in NAFLD, NASH, and fibrosis	Expression in disease	References
SRA	FoxO1 PPARγ	Promotes adipocytes differentiation Promotes insulin-stimulated glucose absorption Prevents FFA oxidation	Upregulated	[36, 37, 39]
NEAT1	miR-146-5p ROCK1	Promotes adipogenesis, lipogenesis, and lipid absorption	Upregulated	[40, 42, 43, 45]
MALAT1	CXCL5	Contributes to the hepatic insulin resistance	Upregulated	[54]
UC372	miR-195/miR-4668 RALGAPA1	Initiates hepatic steatosis	Upregulated	[56, 142]
lncARSR	YAP1	Modulates cholesterol metabolism	Upregulated	[61]
APOA4-AS	HuR protein	Enhances insulin and triglyceride secretion Inhibits gluconeogenesis	Upregulated	[66, 67]
lnc-H19	hnRNP1 PPARγ	Facilitates lipid accumulation in hepatocytes Enhances hepatic steatosis development	Upregulated	[69, 74, 76]
NONMMUT010685 NONMMUT050689	XBP1 RIPK1	Progresses NASH development	Upregulated	[79]
RUNX1	CCL2 PIK3CA	Increases inflammation in liver	Upregulated	[88–90]
HOTAIR	miR-29b PTEN	Accelerates liver fibrosis and carcinogenesis	Upregulated	[95, 143]
Gm15622	miR-742-3p	Increases lipid accumulation in liver	Upregulated	[96]
HULC	MAPK	Promotes NAFLD development	Upregulated	[99, 100]
lnc18q22.2	BCL2 family	Decreases cell viability in hepatic cell lines	Upregulated	[103]
lncLSTR	Cyp8b1 FXR	Enhances TG clearance	Downregulated	[104]
Mirt2	miR-34a-5p	Impedes insulin resistance and hepatic steatosis Modulates gluconeogenesis/lipogenesis in hepatocytes	Downregulated	[109, 115]
MEG3	LRP6 miR-136 Nrf-2 miR-140-5p	Inhibits lipid generation and secretion Promotes osteogenesis despite adipogenesis	Downregulated	[117, 120, 122]
FLRL2	Arntl	Alleviates NAFLD and steatosis Decrease ER stress and liver inflammation	Downregulated	[123]
lncSHGL	hnRNPA1 Calmodulin	Suppresses gluconeogenesis Attenuates hyperglycemia and fatty liver	Downregulated	[125]
lncHR1	FAS SREBP-1c	Inhibits the accumulation of fatty acids and TG	Downregulated	[128, 129]
MRAK052686	ZBTB20	Regulates cellular stress	Downregulated	[132, 133, 135]

## Potentiating role of lncRNAs in NAFLD

### Steroid receptor RNA activator (SRA)

#### Characteristics

Initially, the steroid receptor RNA activator (SRA) was described as a lncRNA that acts as an RNA co-activator to increase the steroid-dependent gene expression of nuclear receptors [29]. SRA plays an essential role in steroidogenesis, myogenesis, cardiomyopathy, and tumorigenesis [29–34]. The *SRA1* gene also creates an alternate transcript encoding a protein known as SRAP, whose function is mostly unknown [35]. It has recently been proven that SRA promotes the differentiation of adipocytes and the absorption of insulin-stimulated glucose in vitro through multiple mechanisms, such as the coactivation of peroxisome proliferator-activated receptor-γ (PPAR-γ), the inhibition of inflammatory genes linked to adipocytes, and the promotion of insulin receptor expression [36, 37]. Normal lipid metabolism in the liver depends on several functionally organized physiological processes that are tightly regulated by genes, especially adipose triglyceride lipase (*ATGL*). ATGL is the most essential and major hepatic triacylglycerol hydrolase [38].

#### Correlation to NAFLD

In a study examining the SRA role in NAFLD in SRA-knockout mice, the results have shown that the deficiency of SRA upregulates the hepatic *ATGL* expression. Moreover, SRA prevents oxidation of FFA by suppressing expression of *ATGL in hepatocytes*. SRA inhibits the transcriptional activity of the fork-head box protein O1 (FoxO1) through an insulin-independent pathway, which subsequently decreases the expression of *ATGL* (a FoxO1 downstream gene), and then reduces FFA β-oxidation in hepatocytes [39]. It is worth mentioning that PPARγ is another target of SRA that promotes the transactivation of *ATGL* [39].

### Nuclear enriched abundant transcript 1 (NEAT1)

#### Characteristics

Nuclear enriched abundant transcript 1 (NEAT1), a nuclear lncRNA, is upregulated in adipocytes and crucial for the development of paraspeckles and is involved in inflammation and lipid absorption by macrophages. NEAT1 plays an integral part in adipogenesis, including oxidation of LDL, lipolysis, and lipid absorption [40–43]. The axis of mTOR-S6K1 plays a vital role in cell growth, proliferation, and differentiation by regulating lipid metabolism and protein synthesis [44].

#### Correlation to NAFLD

The NEAT1 and the mTOR signaling pathway proteins have been amplified in NAFLD in vivo and in vitro. In the meantime, NEAT1 knockdown has decreased the NAFLD through the mTOR/S6K1 pathway, which modulates protein and lipid biosynthesis and remit NAFLD. Additionally, NEAT-1 levels have impacted the mTOR/S6K1 pathway activation [41]. It has been reported that miR-140 is essential for adipogenesis, and NEAT1 has a specific binding site for this miRNA. The interaction of miR-140 and NEAT1 results in enhanced expression and stability of NEAT1. In addition, evidence has indicated that adipogenesis requires miR-140-dependent activation of NEAT1 [43]. Besides, an investigation has revealed that there is a binding between NEAT1, mir-146-5p, and ROCK1 (a pro-apoptotic stimulus). In this regard, it has been shown that NEAT1 promotes ROCK1 expression via downregulating miR-146a-5p. Therefore, NEAT1 increases steatosis by up-regulation of ROCK1 and sponging miR-146a-5p [45].

A water-glycerol transporter called AQP-7 (Aquaporin-7) is essential in suppressing triglyceride synthesis in the Hep-G2 cell line [46]. AQP-7 is induced by 17β-estradiol (E2) through an estrogen receptor. ERα is considered as the leading cause of E2-mediated lipid metabolism and inhibition of steatosis in HepG2 cells. ERα silencing or inhibition could impact NEAT1. Hence, it is proposed that E2 treatment can be considered a possible therapeutic strategy for preventing obesity [42, 46].

### Metastasis-associated lung adenocarcinoma transcript 1 (MALAT1)

#### Characteristics

MALAT1 is a long length lncRNA that contains more than 8000 nucleotides, which is upregulated in diabetic mice. Recently the role of MALAT1 in the development of diabetic complications has received attention. MALAT1 dysregulation is implicated in the pathogenesis of diabetes-associated retinopathy and microvascular disease. Furthermore, MALAT1 induces the expression of inflammatory cytokine in high glucose-treated endothelial cells. The deletion of MALAT1 impedes liver cells' development, indicating MALAT1 contributes to hepatic insulin resistance [47–50].

#### Correlation to NAFLD

The expression of MALAT1 is upregulated in the hepatocyte of the animal model of type-2 diabetes (ob/ob mice) upon palmitate exposure. Apart from the elevated MALAT1, palmitate therapy results in decreased mRNA and nuclear sterol regulatory element-binding protein (SREBP)-1c concentrations [51]. SREBP-1c, which abundantly expresses in hepatocytes, is accumulated in the liver of diabetic by insulin [52, 53]. It has been identified that CXCL5 has been introduced as a MALAT1 target in hepatocytes. Enhanced levels of CXCL5 transcription and protein were identified in the fibrotic liver. Data has shown that the knockdown of MALAT1 decreases the mRNA and protein level of CXCL5 in Hep-G2 cells [54].

### Ultra-conserved element (UC372)

#### Characteristics

UC372 comprises one of the ultra-conserved lncRNA with 100% identity across the rat, mouse, and human genomes [55]. This gene has been located in a cluster that developmental genes and transcription factors encode.

#### Correlation to NAFLD

UC372 has been upregulated in a murine model of type-2 diabetes mellitus (db/db mice), high-fat diet (HFD-fed) mice, and NAFLD patients, which proposes the role of this lncRNA in liver steatosis and fatty liver [56]. It has been suggested a mechanism that UC372 initiates hepatic steatosis through the prevention of miR-195/miR-4668 related target gene, including acetyl-CoA carboxylase (*ACC*), fatty acid synthase (*FASN*), stearoyl-CoA desaturase 1 (*SCD1*), and lipid uptake related genes such as CD36, leads to the accumulation of hepatic lipids [56]. Such data indicate that hepatic steatosis is specifically caused by overexpressed hepatic UC372.

### LncRNA activated in RCC with sunitinib resistance (lncARSR)

#### Characteristics

LncARSR is a recently identified lncRNA with 591 length nucleotides. The major studies about lncARSR have been done in cancer, especially in hepatocellular carcinoma and renal cell carcinoma [57, 58].

#### Correlation to NAFLD

In terms of fatty liver disease, it has been clarified that lncARSR levels are significantly elevated in the liver and serum of patients suffering from NAFLD and in the liver of MCD (methionine-choline deficient) mice compared to chow diet-fed mice [59]. By conducting in vitro study, it has been confirmed that lncARSR overexpression induces the expression of lipogenic genes like *SREBP-1c*, *SCD1*, *FASN* [59]. Moreover, through Akt/SREBP-1c pathway, lncARSR controls hepatic lipogenesis, which provides new evidence of the metabolic role of lncARSR [59].

In both human hypercholesterolemia and high-cholesterol diet mice, the expression of lncARSR was increased. The knockdown of lncARSR in a murine model and Hep-G2 cell line has been shown that cholesterol metabolism is modulated by lncARSR in vitro and in vivo [60]. Li et al. stated that lncARSR modulates hepatocellular carcinoma resistance to doxorubicin via PTEN‐PI3K/Akt pathway [58]. On the other hand, LncARSR specifically binds and blocks YAP1 phosphorylation and encourages YAP1 to be imported into the nucleus [61]. Blockade of YAP1 phosphorylation causes the activation of YAP1. It has been reported that the YAP signaling pathways promote the progression and development of NAFLD [62].

### Apolipoprotein A4 Antisense (APOA4-AS)

#### Characteristics

Apolipoprotein A4, as a plasma protein, regulates many metabolic pathways, including glucose and lipid metabolism [63]. Primarily, hepatocytes and the small intestine synthesize APOA4 and secrets into the blood. The mutations in APOA4 has been correlated with an altered level of plasma lipid [64]. Moreover, APOA4 enhances TG secretion and insulin production, inhibits gluconeogenesis, and as a result, is linked to type 2 diabetes and obesity [65, 66]. APOA4-AS, as a reverse-transcribed of *APOA4* gene, has been considered regulatory lncRNA of *APOA4*.

#### Correlation to NAFLD

In vitro and in vivo studies have shown that APOA4-AS is crucial to maintain *APOA4* expression. Therefore, knockdown of APOA4-AS in hepatocytes leads to reduced mRNA level of *APOA4* and plasma triglyceride and TC in ob/ob mice, which proposes a stabilizing role of APOA4-AS for APOA4 [67].

An RNA-binding protein called human antigen R (HuR) is the key to solve puzzles and target proteins in the APOA4-AS mechanism of action. The HuR protein modulates mRNA stability and translation efficacy, which has a central role in the proliferation, growth, and survival of cells [68]. There is a two proposed HuR-binding site in the structure of APOA4-AS. Overall, these findings suggest that HuR is a key stabilizing protein for APOA4-AS and APOA4. HuR is recruited to APOA4-AS and APOA4 complex [67].

### lncRNA H19

#### Characteristics

H19, as one of the foremost identified lncRNAs, has many physiological and pathological effects on the stability of mRNAs [69]. The diminished level of H19 expression in the adult liver compared with the fetal liver has proposed its regulatory function in hepatic metabolism [70]. As mentioned earlier, hnRNPA1 is an RNA binding protein that can regulate pre-mRNA splicing, mRNA stability, cell programming, and tumor progression [71–73].

#### Correlation to NAFLD

In terms of NAFLD, the action mechanism of H19 relies on hnRNPA1. It has demonstrated that the interaction of H19 and hnRNPA1 under fasting conditions enhances nuclear mRNA translocation and protein levels of SREBP1. Also, prolonged-expression of H19 facilitates lipid accumulation in hepatocytes, enhances hepatic steatosis development, and metabolic pathway disruption. On the other hand, fatty acids stimulate the expression of hnRNPA1 and H19, which indicates being of positive feedback between fatty acid input and lncRNA H19 expression [74].

Another action mechanism of H19 relies on the PPARγ/miR-130a axis. PPARγ is a highly-expressed nuclear receptor in adipose tissue that its upregulation and elevated activity have been observed in NAFLD patients [75]. It has been found that H19 knockdown inhibits the expression of PPARγ, which results in the upregulation of miR-130a, and is considered an attenuating agent of NAFLD through inducing apoptosis in hepatic stellate cells [76]. As a result of the interplay between lncRNA H19, hnRNPA1 protein, PPARγ, and miR-130a, it can be concluded that H19 is one of the most important lncRNAs in the formation of fatty liver and steatosis. These findings have suggested targeting of lncRNA H19 to overcome NAFLD.

### LncRNA NONMMUT010685 and NONMMUT050689

#### Characteristics

ATP citrate lyase (ACLY), by converting citrate to acetyl-CoA, plays a crucial step in fatty acid biosynthesis and links this cycle to carbohydrate metabolism in animals [77, 78]. In NAFLD samples, the ACLY enzyme is increased significantly [79]. In addition, the ACLY activity has been associated with metabolic disorders such as hepatic steatosis, dyslipidemia, and reduced tolerance to glucose [78, 80]. A study has proposed that in leptin-deficient mice and the *ACLY* knockdown model, plasma TG and VLDL levels have been decreased [81].

#### Correlation to NAFLD

It has been reported that lncRNAs NONMMUT010685 and NONMMUT050689 have been increased in NAFLD samples. These two lncRNAs have been proposed as regulators of *XBP1* and *RIPK1*, respectively [79]. Lee et al. and Kaser et al. have suggested that XBP1, as a key regulator of unfolded proteins, has important significance for human dyslipidemias and is crucial for the maintenance and development of secretory cells, which is associated with JNK activation [82, 83]. Failure to effectively degrade proteins in response to XBP1 activation have been posed NASH patients at high risk for progression to cirrhosis [84].

RIPK1, which is associated with the inflammation and cell death pathways, is reported to initiate RIPK3-mediated necroptosis by its kinase activity. During NASH development, RIPK1 limited the progression of liver fibrosis in hepatocytes [85, 86]. It has been shown that the XBP1 and RIPK1 are downregulated in NASH, indicating the involvement of XBP1 and RIPK1 in NASH pathogenesis [79]. Altogether, the upregulation of lncRNAs NONMMUT010685 and NONMMUT050689 in NAFLD downregulates XBP1 and RIPK1 and consequently increases ACYL enzyme and progresses development of NASH.

### Runt-related transcription factor 1 (RUNX1)

#### Characteristics

The central mechanisms for the NAFLD progression to NASH, cirrhosis, and hepatocellular carcinoma are inflammation and oxidative stress-derived pathological angiogenesis [87]. Since RUNX1 has been proposed as a regulator for inflammation (TLR4-mediated inflammation), angiogenesis (via VEGF), and hematopoiesis, this lncRNA has been investigated for its role in NAFLD [88, 89].

#### Correlation to NAFLD

The expression of RUNX1 (also defined as acute myeloid leukemia 1 (AML1)) has correlated with the severity of NAFLD. The qRT-PCR and immunohistochemistry (IHC) analyses have shown that RUNX1 and its target gene, including *CCL2* and *PIK3CA*, positively correlate to steatosis, fibrosis, and inflammation grade [90]. Knockdown of RUNX1 in the HUVEC cell line has affected mRNA expression of angiogenic and chemotactic factors and adhesion molecules such as VEGF, CCL2, PECAM1, and VCAM1. So RUNX1 has been shown that increases the angiogenic activity of the HUVECs cell line [90].

Altogether, the action mechanism of RUNX1 in liver-related disease relies on the upregulation of its downstream genes, including VEGFs, chemokines, and adhesion molecules. Therefore, it has been suggested that RUNX1 targeting can overcome the exacerbation of fatty-acid related liver diseases.

### Homeobox transcript antisense RNA (HOTAIR)

#### Characteristics

Phosphatase and tensin homolog (PTEN), an enzyme linked to cellular growth and negative regulator of insulin receptor signaling, have been involved in the NAFLD [91, 92]. It has been shown that the null mutation of *PTEN* results in triglyceride accumulation and steatohepatitis, hepatomegaly, and later conversion to hepatocellular carcinoma [93]. Moreover, the expression of *PTEN* is downregulated in hepatocytes exposed to FFAs via NF-κB/mTOR dependent pathway, which may cause hepatic steatosis [94].

#### Correlation to NAFLD

HOTAIR is a lncRNA that its upregulation has been shown in the liver fibrosis, which causes acceleration of carcinogenesis in HBV-infected liver [95]. siRNA-mediated knockdown of HOTAIR inhibits PTEN downregulation and accumulation of triglyceride in FFA-treated HepG2 cells. The upregulation of HOTAIR has also been induced upon FFA-treated HepG2 cells via NF-κB signaling. In addition, the withdrawal of the FFAs treatment disappears the effects of the HOTAIR and PTEN expressions. These findings indicate that HOTAIR has negative impacts on PTEN. The downregulation of miR-29b is a proposed action mechanism of HOTAIR on PTEN. Since HOTAIR has a binding site for miR-29b, it has been suggested that HOTAIR by sponging miR-29b leads to enhanced methylation of PTEN and progression of hepatofibrosis [95].

### lncRNA Gm15622

#### Characteristics and correlation to NAFLD

In the liver of high-fat diet obese, ob/ob, and db/db mice, Gm15622 has highly upregulated. In vitro study showed that the upregulation of Gm15622 increases lipid accumulation while Gm15622 silencing reduces lipid accumulation in AML12 (alpha mouse liver 12) cell line [96]. Similar to several mentioned studies, Gm15622 modulates SREBP-1c through miR-742-3p sponging. It has been proposed that Gm15622 has a binding site for miR-742-3p. Since miR-742-3p has been identified as a negative regulator of SREBP-1c, Gm15622 by sponging this miRNA and subsequently, SREBP-1c protein enhancement is involved in NAFLD progression [96].

Moreover, via the siRNA-dependent knockdown of Gm15622, it has been shown that Gm15622 regulates the FAS enzyme [96]. As a first-line medication for type-2 diabetes treatment, metformin has an alleviating effect on NAFLD [97]. It has been suggested that metformin reduces expression of SREBP-1c, Gm15622, and FAS while increases miR-742-3p level and therefore contributes to NAFLD improvement [96].

### Highly upregulated in liver cancer (HULC)

#### Characteristics

HULC has currently been proposed to be implicated in the development, cell proliferation, and chemoresistance of HCC [98, 99].

#### Correlation to NAFLD

The increased level of HULC expression is verified in the hepatocytes of NAFLD rats. HULC inhibition reduces hepatocyte apoptosis and improves hepatic fibrosis rates and lipid deposition in NAFLD rats' liver [100]. The action mechanism of HULC depends on MAPK (p38/JNK) signaling pathway. JNK, a member of the MAPK family and is stimulated by FFA, inflammation, oxidative, and reticulum endoplasmic stress, is involved in NAFLD's pathogenesis [101, 102]. The inhibition of HULC could lead to the blockade of the MAPK signaling pathway in the liver of NAFLD rats. Since the inhibition of HULC could inhibit NAFLD progression, it can serve as a novel target for NAFLD treatment [100].

### lnc18q22.2

#### Characteristics

It has been discovered that lnc18q22.2 is a liver-specific lncRNA, which is crucial for growth, mRNA translation, cell death, apoptosis, oxidation–reduction process, and viability of hepatocytes. The level of lnc18q22.2 expression is increased in the liver biopsy of patients with steatohepatitis [103]. In addition to the specified expression of lnc18q22.2 in the liver, RT-PCR analysis has shown that lnc18q22.2 was expressed in liver cell lines included Hep3B, Huh7, IHH, HepG2, and primary human hepatocytes compared with HEK293T and HeLa cells [103].

#### Correlation to NAFLD

The knockdown of lnc18q22.2 leads to a decreased cell viability or lethal phenotype in hepatic cell lines. The data indicate that lnc18q22.2 negatively regulates genes that are involved in the process of oxidation–reduction. The elevated level of lnc18q22.2 expression emphasizes a putative suppression effect on redox-reactions genes [103]. The knockdown of lnc18q22.2 downregulates anti-apoptotic genes, including BCL2 family proteins. These effects leave behind a necrosis-like phenotype in the liver, which can be concluded that has resulted from lnc18q22.2 knockdown. Altogether, it can be claimed that lnc18q22.2 may introduce a new therapeutic target of NASH treatment [103].

## Preventing role of lncRNAs in NAFLD

### Liver-specific triglyceride regulator (lncLSTR)

#### Characteristics

In a mouse genome region syntenic to human chromosome 1q25, lncLSTR is a liver-specific and intergenic lncRNA, which is considered a potential metabolic regulator in animals [104].

#### Correlation to NAFLD

It has been demonstrated that lncLSTR knockdown reduced the level of triglyceride in mice. Furthermore, the depletion of lncLSTR increases lipoprotein lipase (LPL) activities, upregulates the expression of apolipoprotein C2 (apoC2), and leads to enhanced plasma triglyceride clearance. In a "rescue" experiment in which lncLSTR expression level significantly increased compared to lncLSTR-depleted mice, it has been clarified that there is a relation between lncLSTR and elevated level of apoC2 and LPL.

Farnesoid X receptor (FXR)-mediated pathway has been proposed as a regulatory mechanism of lncLSTR [104]. FXR is considered the primary bile acid receptor (BAR) in the liver and one of the well-known regulators of apoC2 expression, which is involved in glucose and lipid metabolism [105, 106]. Firstly, FXR knockdown resulted in efficiently blunted the lipid-lowering effect of lncLSTR depletion in mice; secondly, diminished apoC2 expression in lncLSTR-depleted mice. These findings confirm the theory that the enhanced TG clearance in lncLSTR knockdown mice depends on FXR activity and the increased expression of apoC2 [104].

Cytochrome P450 Family 8 Subfamily B Member 1 (Cyp8b1) is an important enzyme in the bile acid synthesis pathway, which determines the ratio of muricholic acid (MCA) and cholic acid (CA) as the two most abundant bile acids in the mouse [107, 108]. Cyp8b1 reduction in primary hepatocytes and lncLSTR-depleted mice's livers, results in a substantial change in bile acid composition. The altered bile acid composition triggers FXR signaling to elevate apoC2 levels, leading to enhanced TG clearance in mice [104].

### Myocardial Infarction Associated Transcript 2 (Mirt2)

#### Characteristics

Mirt2 has been introduced as an inflammation-suppressor lncRNA which its overexpression protects mice from endotoxemia and multi-organ dysfunction by inhibition of TRAF6 through K63 ubiquitination. This process subsequently prevents inflammatory reactions [109]. These findings can address the inhibitory effect of Mirt2 on NAFLD, which per se count as an inflammatory condition. Ubiquitin-specific peptidase 10 (USP10) is a protease that acts as an anti-stress factor and tumor-suppressor enzyme during cancer and regulates cellular metabolism.

#### Correlation to NAFLD

USP10 deletion leads to a significant increase in lipid droplets' formation, production of lactate, and expression of glycolytic genes [110–113]. It has been demonstrated that USP10 inhibits hepatic steatosis and insulin resistance via Sirt6, in which Sirt6 represses the transcription levels of *SREBP1*/*SREBP2* and their target genes [114]. Collectively, in livers of the obese and fasting mouse, the expression of Mirt2 was decreased. In contrast, Mirt2 knockdown promotes hepatic steatosis and insulin resistance. In an investigation for the Mirt2-associated molecular mechanism, it has been emphasized that miR-34a-5p is considered the target of Mirt2, and miR-34a-5p is a repressor of USP10. When Mirt2 inhibits miR-34a-5p subsequently increases USP10 activity, which modulates gluconeogenesis/lipogenesis in hepatocytes. Finally, Mirt2 prevents the formation of fatty liver [115]. Taken together, Mirt2 overexpression may be beneficial for NAFLD treatment.

### lncRNA maternally expressed gene 3 (MEG3)

#### Characteristics

By observing the downregulation of MEG3 (also known as gene trap locus 2 (GTL2)) in human and mouse fatty liver tissues, it has been hinted that MEG3 might play an underlying role in the NAFLD progression [116]. MEG3 is also reported to be able to suppress fatty acid deposition [117]. There are some proposed mechanisms for MEG3 action in NAFLD.

#### Correlation to NAFLD

Low-density lipoprotein receptor-related protein 6 (LRP6) is a well-established lipid generation and secretion regulating factor via AKT/mTOR pathway. It has been demonstrated that decreased MEG3 level is associated with decreased LRP6 protein levels simultaneously [118]. It is worth noting that the AKT/mTOR pathway mediates various lipid metabolic genes. On the other hand, miR-21 is a regulator of LRP6, MEG3, and LRP6. Therefore miR-21 has been introduced as a cholesterol and TG metabolism regulator [119]. The overexpression of MEG3 resulted in the suppression of miR-21, which led to LRP6 potentiation for inhibition of the AKT/mTOR pathway [120].

Another proposed action mechanism of MEG3 relies on miR-136 and nuclear factor erythroid 2–related factor 2 (Nrf2). Nrf2 is a basic leucine zipper protein that regulates antioxidant proteins' expression and helps NAFLD regression in response to antioxidants [121]. miR-136 is upregulated in NAFLD mice and downregulated in the liver of antioxidant-treated mice. It has been suggested that MEG3 is a downstream target of miR-136. The increased level of miR-136 and low levels of MEG3 and Nrf2 were involved in NAFLD development [122].

In addition to NAFLD regression, the role of MEG3 has been more pronounced in the differentiation of human adipose-derived stem cells (hADSC) to the osteogenesis via regulation of miR-140-5p. There is evidence that MEG3 competes with miR-140-5p to inhibit hADSC differentiation to adipocytes [117].

### Fatty liver-related lncRNA 2 (FLRL2)

#### Characteristics

Via a genome-wide lncRNA microarray, FLRL2 has been considered a potential key regulator in the rodent model of NAFLD. In terms of histological distribution, FLRL2 is widely detectable in several tissues such as the liver, adipose tissue, spleen, pancreas, and even brain tissue [123].

#### Correlation to NAFLD

There is a compelling reason that FLRL2 overexpression alleviates NAFLD and steatosis and vice versa [123]. FLRL2 has been located on the intronic region of aryl-hydrocarbon receptor nuclear translocator-like (*Arntl*), and *Arntl* is considered an FLRL2 cis-target [124]. The expression of *Arntl* in NAFLD mice is inhibited, and its overexpression relieves cellular steatosis and ameliorates NAFLD. On the contrary, the siRNA-dependent knockdown of *Arntl* leads to the aggravation of NAFLD [123]. Since the expression pattern of *Arntl* and FLRL2 is similar, it has been proposed that the Arntl-Sirt1 axis has been considered the FLRL2 mechanism of action [123]. The expression of FLRL2 in NAFLD is inhibited, and on the contrary, the endoplasmic reticulum stress, hepatic inflammation, and lipogenesis are stimulated. In this condition, the Arntl transcription is promoted by FLRL2, and transcribed Arntl proteins reenter the nucleus and bind to the promoter of Sirt1, and enhances its transcription. This process alleviates lipogenesis, endoplasmic reticulum stress, and hepatic inflammation by inhibiting inflammatory gene transcription, including *ACC*, *LPL*, *SREBP1*, *FAS*, *CHOP*, and *TNFA* [123]. These findings potentiate the therapeutic target of FLRL2 in NAFLD.

### lncRNA suppressor of hepatic gluconeogenesis and lipogenesis (lncSHGL)

#### Characteristics

LncSHGL, known as specific lncRNA of the liver, has been demonstrated to be reduced in obese mouse livers. Moreover, human homologous of lncSHGL named lncRNA B4GALT1-AS1 was also decreased in human livers with steatosis [125].

#### Correlation to NAFLD

Overexpression of hepatic lncSHGL suppresses gluconeogenesis and attenuates fatty liver and hyperglycemia in HFD mice, while hyperglycemia and lipid accumulation in normal mice is induced by lncSHGL repression. The action mechanism of lncSHGL is dependent on hnRNPA1 [125].

The primary action mechanism of lncSHGL relies on the Calmodulin (CaM) protein level. CaM, which regularly represses mTOR and activates the Akt pathway, per se inhibits obesity in mice. It has been reported that lncSHGL recruits hnRNPA1 to increase the efficacy of CaM mRNA translation [126, 127]. Altogether, the lncSHGL/hnRNPA1/CaM axis plays a critical role in suppressing hepatic lipogenesis and gluconeogenesis. It has been suggested that short time repression of the lncSHGL/hnRNPA1/CaM axis is probably advantageous for increased fasting gluconeogenesis and can be a strategy for type-2 dialethic Mellitus and steatosis treatment [125].

### lncRNA HCV regulated 1 (HR1)

#### Characteristics

Lnc-HR1 was firstly reported as being upregulated in hepatitis C (HCV)-infected Huh7 cells. In the transgenic mice model, lncHR1 have demonstrated regulatory function on lipid accumulation via SREBP-1c [128]. Overexpression of lncHR1 prevents the expression of *FAS* and *SREBP1* and subsequently inhibits the accumulation of lipid droplets-containing oleic acid and TG in the liver [128].

#### Correlation to NAFLD

The expression of lncHR1 leads to less hepatic expression of *ACC*, *FAS*, *SREBP1*, and reduced levels of hepatic and plasma TG after a high-fat diet in mice [128]. The molecular mechanism behind the SREBP-1c regulatory function relies on PDK1/AKT/FoxO1 signaling pathway. This pathway's activation results in increased glucose uptake and glycolysis and, consequently, de novo lipid synthesis [129]. It has been hypothesized that SREBP-1c activation is done by this pathway. Therefore, it is concluded that PI3K/AKT signaling upregulates SREBP-1c and promotes fatty acid synthesis [130, 131]. Thus, the upregulation of lnc-HR1 could result in NAFLD regression.

### LncRNA MRAK052686

#### Characteristics

MRAK052686 is a conserved lncRNA located around *ZBTB20* (an important glucose homeostasis regulator and related to liver dysfunction) gene and has been strongly correlated with the function of antioxidant factor Nrf2 [132, 133].

#### Correlation to NAFLD

MRAK052686 is co-expressed with other genes related to NAFLD, such as the fatty acid-binding proteins *Gcs1* and *Fabp7*, which are implicated in ER protein processing [134]. Taking all together, these findings propose that the lncRNA MRAK052686 may perform pivotal roles in NAFLD by affecting ER-related genes that regulate cellular stress responses [133].

It has been demonstrated that MRAK052686 and its associated gene *Nrf2* are downregulated in the NASH. Berberine is a botanic compound extracted from the traditional Chinese herb Rhizoma Coptidis to treat inflammatory diseases [135]. There is a piece of evidence that Berberine alleviates NAFLD by modulation of lncRNA MRAK052686 and its associated gene *Nrf2* and the reduction of ER-related stress [133].

## Other important but lesser-known lncRNAs in liver steatosis and fibrosis

In NCTC1469 cells, a cellular model of NAFLD, the microarray has shown that lncRNA-AK012226 has upregulated. siRNA-dependent knockdown of lncRNA-AK012226 has revealed that there is a link between NAFLD and lncRNA-AK012226. In addition, knockdown of lncRNA-AK012226 results in decreased lipid accumulation in free fatty acid-treated NCTC cells, which proposes this lncRNA's functional role in NAFLD pathogenesis. Nevertheless, the underlying molecular mechanism of lncRNA-AK012226 has not yet been elucidated in regulating lipid accumulation and NAFLD pathogenesis [136].

Alu-mediated p21 transcriptional regulator (APTR) has been addressed to have essential roles in cell cycle regulation. This lncRNA has been upregulated in fibrotic liver samples and has a putative function in liver fibrogenesis. The knockdown of APTR inhibits collagen accumulation through the abrogation of TGF-β-dependent upregulation of α-SMA, in vivo [137, 138].

lncRNA-NR002155.1 has been identified in the liver tissue of carbon tetrachloride (CCI4; a hepatotoxic substance)-treated mice amongst 231 examined lncRNAs. The downregulation of lncRNA-NR_002155.1 has been found in fibrotic tissue and has been demonstrated to have a putative role in NAFLD [139].

LncRNA liver fibrosis-associated lncRNA 1 (LFAR1) has been firstly introduced in an investigation for the study of lncRNA in hepatofibrosis. LFAR1, a liver-enriched lncRNA, binds to *Smad2/3* and promotes the transcription of genes involved in liver fibrosis, including *Smad2/3*, *Notch2/3*, and *TGFB*. Therefore, this lncRNA activates TGFβ/Notch signaling pathway and promotes liver fibrosis in HFD mice [140].

TGFB2-OT1 and RP11-128N14.5 have been introduced in patients with fibrosis stages 3–4 and NAFLD activity score > 5, respectively. It has been proposed that these two lncRNAs are involved in the severity of liver steatosis and fibrosis. Moreover, it has been claimed that TGFB2-OT1 could improve advanced fibrosis discrimination [141].

Plasmacytoma variant translocation 1 (PVT1), whose role was more pronounced in several cancers, was also shown to contribute in fibrotic liver tissues via downregulation of *PTCH1* expression and positive regulation of the Hedgehog pathway. These mechanisms are vital in collagen deposition and liver fibrosis [141].

## Conclusion and future directions

NAFLD has increasingly become prevalent around the world, particularly in Western countries. It is the most prevalent form of chronic liver disease so that it impacts about one-quarter of the U.S. population. Sometimes people suffering from NAFLD may develop an aggressive form of fatty liver disease called NASH, characterized by liver inflammation, which is likely to progress towards progressive liver failure known as cirrhosis. NAFLD's clinical complexity and pathophysiology have necessitated a great variety of potential biomarkers for a specific diagnosis, prediction, and treatment of the disease. NAFLD and NASH classification is usually achieved by evaluating various clinical, biochemical, imaging procedures, blood biomarkers, and liver biopsy. The discrimination of NAFLD and NASH by the mentioned-procedure is not still precise and needs to be collected more data to diagnose definitely. Considerable evidence points to the potential effects of lncRNAs in regulating gene expression, offering new opportunities to comprehend the course of NAFLD. Indeed, several lncRNAs have been expressed differently in NAFLD patients compared with a healthy population, and some lncRNAs through various mechanisms have been involved in NAFLD pathogenesis.

The role of lncRNAs in fatty liver and hepatic steatosis has attracted much attention during the last decade. Since some lncRNAs, including NEAT1, RUNX1, and SRA, have been increased, and some lncRNAs including, MEG3, FLRL2 are decreased in NAFLD, they can be considered as a molecular diagnostic panel for NAFLD diagnosis. However, more focus is needed in research investigating lncRNAs in NAFLD, particularly for validation, before the results can be translated into clinical uses.

There is no certain cure for NAFLD and NASH, and routine treatments are a low-fat diet, weight loss, and diabetes control. In terms of treatment, there is also a possibility to target lncRNAs for therapeutic approaches. Hence, the direct targeting of a single or a set of lncRNAs conceivably leads to the modulation of NAFLD. The inhibition or mimicking of lncRNAs is one of the promising approaches in NAFLD's targeted therapy. Mimicking is an approach for the re-expression of downregulated lncRNA. On the contrary, the inhibition approaches, including antisense and RNA interference (RNAi), are used to silence upregulated lncRNAs to prevent the pathological process. Therefore, designing a panel of inhibitory and stimulating lncRNAs in NAFLD, it is hoped that these approaches might be promising for treating NAFLD and NASH.

Most studies regarding the relationship between NAFLD and lncRNAs have been investigated in vitro and limited animal studies. Even though encouraging, studies have not yet been developed to investigate the above-listed strategies for clinical translation, mainly due to the lack of an actual amount of observations that provide conclusive deductions regarding the role of lncRNAs in NAFLD development in vivo, especially in human. Therefore, the exact mechanisms of various lncRNAs on NAFLD and NASH development are needed to be clarified, at least in animal models. The main underlying mechanisms, including RNA and protein interaction with disease-specific lncRNAs and sponging targets, have to be discovered comprehensively. Another remaining challenge will be to enhance the techniques for lncRNA detection and identification in related tissues and biofluids, which may consequently defend the potential clinical values of lncRNAs as biomarkers.

In conclusion, lncRNAs are newly established as essential regulators in a variety of biological processes. Understanding their role in humans, especially inflammation-related diseases, has revealed novel considerable knowledge that can be applied to develop innovative diagnostic and therapeutic approaches. While recent advances in lncRNA studies of NAFLD indicate improvement toward incorporating lncRNAs into the pre-existing miRNA-mRNA-protein regulatory network, several pressing issues remain. Taking all together, further researches is required to explain the role of lncRNAs in NAFLD pathophysiology and discover their applicability as therapeutic targets or invasive diagnostic markers.

## Data Availability

Not applicable.

## Abbreviations

ACC: Acetyl–coenzyme A carboxylase enzyme
ACLY: ATP citrate lyase
ADSC: Adipose-derived stem cells
AML1: Acute myeloid leukemia 1
AML12: Alpha mouse liver 12
APOA4: Apolipoprotein A4 Antisense
APTR: Alu-mediated p21 transcriptional regulator
AQP: Aquaporin
ARSR: Activated in RCC with sunitinib resistance
ATGL: Adipose triglyceride lipase
BCL2: B-cell lymphoma 2
BIP: Binding immunoglobulin protein
CCL2: CC chemokine ligand 2
CHOP: C/EBP homologous protein
CXCL5: CXC chemokine ligand 5
ER: Endoplasmic Reticulum
FASN: Fatty acid synthase
FFA: Free fatty acid
FLRL2: Fatty liver-related lncRNA 2
FXR: Farnesoid X receptor
GTL2: Gene trap locus 2
GWAS: Genome-wide association studies
HCC: Hepatocellular carcinoma
HCV: Hepatitis C virus
HFD: High-fat diet
HMGCR: Hydroxy-3-Methylglutaryl-CoA Reductase
HOTAIR: Homeobox transcript antisense RNA
HR1: HCV regulated 1
HULC: Highly upregulated in liver cancer
HUVEC: Human umbilical vein endothelial cells
IHC: Immunohistochemistry
IHH: Indian Hedgehog
INSM2: Insulinoma-associated 2
IRE: Iron response element
JNK: Janus Kinase
LDL: Low-density lipoprotein
LFAR1: Liver fibrosis-associated lncRNA 1
LPL: Lipoprotein lipase
LRP6: Low-density lipoprotein receptor-related protein 6
LSTR: Liver-specific triglyceride regulator
MALAT1: Metastasis-associated lung adenocarcinoma transcript 1
MAPK: Mitogen-activated protein kinase
MCA: Muricholic acid
MCD: Methionine-choline deficient
MCP1: Monocyte chemoactive protein 1
MEG3: Maternally expressed gene 3
NAFLD: Non-alcoholic fatty liver disease
NASH: Non-alcoholic steatohepatitis
NEAT1: Nuclear enriched abundant transcript 1
NF-κB: Nuclear factor-kappa B
NRF2: Nuclear factor erythroid 2–related factor 2
PDK1: Phosphoinositide-dependent protein kinase-1
PECAM1: Platelet And Endothelial Cell Adhesion Molecule 1
PI3K: Phosphoinositide 3-kinases
PIK3CA,: Phosphatidylinositol 4,5-bisphosphate 3-kinase
PNPLA3: Patatin-like phospholipase domain-containing protein 3
PPAR: Peroxisome proliferator-activated receptors
PTCH1: Protein patched homolog 1
PTEN: Phosphatase and tensin homolog
PVT1: Plasmacytoma variant translocation 1
RALGAPA1: Ral GTPase Activating Protein Catalytic Subunit Alpha 1
RCC: Renal cell carcinoma
RIPK1: Receptor-interacting serine/threonine-protein kinase 1
hnRNP1: Heterogeneous nuclear ribonucleoprotein A1
ROCK1: Rho-associated kinases 1
RUNX1: Runt-related transcription factor 1
SCD1: Stearoyl-CoA desaturase 1
SHGL: Suppressor of hepatic gluconeogenesis and lipogenesis
α-SMA: Alpha-smooth muscle actin
SRA1: Steroid receptor RNA activator 1
SRAP: Steroid receptor RNA activator protein
SREBP: Sterol regulatory element-binding protein
TC: Total Cholesterol
TG: Triglyceride
TGF: Transforming Growth Factor
TGFB2-OT1: TGFB2 Overlapping Transcript 1
TLR4: Toll-like receptor 2
TM6SF2: Transmembrane 6 superfamily 2
TNF: Tumor necrosis factor
mTOR: Mammalian target of rapamycin
TRAF6: Tumor necrosis factor receptor-associated factor 6
UC372: Ultra-conserved element 372
USF1: Upstream stimulatory factor 1
USP10: Ubiquitin-specific peptidase 10
VCAM1: Vascular cell adhesion protein 1
VEGF: Vascular endothelial growth factor
VLDL: Very low-density lipoprotein
XBP1: X-box binding protein 1
YAP1: Yes-associated protein 1
ZBTB20: Zinc finger and BTB domain-containing protein 20
